# Ketcher: web-based chemical structure editor

**DOI:** 10.1186/1758-2946-3-S1-P3

**Published:** 2011-04-19

**Authors:** B Karulin, M Kozhevnikov

**Affiliations:** 1 SciTouch LLC, St.-Petersburg, Russia

## 

Ketcher is an open-source web-based chemical structure editor developed by SciTouch LLC. Being written on pure JavaScript it incorporates high performance, good portability and light weight. Editor supports late versions of all most popular browsers, such as Internet Explorer, Firefox, Safari, Opera and Chrome.

It is developed with minimum third-party code to remain its light weight. It is platform-independent and can be used as a structure viewer as well. SciTouch keeps on working to add more features to Ketcher in near future.

Ketcher is available under Affero GPL license.

Features:

• Standalone mode. Ketcher supports standalone mode in which no server support is required.

• Scalable Vector Graphics (SVG) for rendering. Ketcher uses SVG to achieve the best quality of in-browser chemical structure rendering. SVG standard is supported by most of modern browsers and provides smooth and light-weight drawing. IE rendering is based on VML (Vector Markup Language) instead.

• SMILES strings. SMILES is a compact format for chemical structure representation. Ketcher gives you ability to load and save structure in this useful format.

• Automatic layout (clean up). Server-side structure layout is implemented basing on Indigo cheminformatics library with a Python wrapper. Indigo provides fast 2D structure representation which satisfies common chemical drawing standards.

• And also: MDL (Symyx) molfiles support, hot keys, stereochemistry support, complete undo/redo history and much more.

Ketcher web site: http://scitouch.net/ketcher

Try Ketcher demo: http://scitouch.net/ketcher-demo/ketcher.html

